# Musculoskeletal ultrasound assessment in pediatric knee hypermobility: a case control study

**DOI:** 10.1186/s12969-021-00546-w

**Published:** 2021-04-29

**Authors:** Laura R. Ballenger, Melissa Moore-Clingenpeel, Edward J. Oberle

**Affiliations:** 1 Department of Rheumatology, Nationwide Children’s Hospital, 700 Children’s Drive, Columbus, OH 43205 USA; 2 Biostatistics Resource at Nationwide Children’s Hospital, Abigail Wexner Research Institute, 700 Children’s Drive, Columbus, OH 43205 USA

**Keywords:** Ultrasonography, Hypermobility, Knee, Tendon hyperemia

## Abstract

**Background:**

While musculoskeletal ultrasound (MSUS) use in pediatric rheumatology is becoming more common, the majority of pediatric MSUS literature continues to focus on ultrasound findings in healthy children and juvenile idiopathic arthritis with little discussion of other musculoskeletal problems that may mimic arthritis such as joint hypermobility. Chronic joint pain related to hypermobility is a common referral to pediatric rheumatology clinics. Our aim is to describe the musculoskeletal ultrasound (MSUS) characteristics of the knee in a population with joint hypermobility and pain in comparison to control participants.

**Methods:**

Participants were recruited into three groups for a case-control study. Case group participants had knee hypermobility and pain symptoms (H + P). Participants in one control group had knee hypermobility without pain symptoms (H-P), and participants in the other control group had no knee hypermobility or pain symptoms (NP). B-mode and Doppler MSUS images were obtained and scored for each knee. Descriptive statistics are used for demographic variables and MSUS findings. Regression analysis is used to evaluate risk of synovial effusion and higher synovial effusion/hypertrophy quantitative score.

**Results:**

MSUS assessment was performed on 91 knees of 50 participants. H + P knees were more likely to have positive findings noted on MSUS (94% vs. 70% of H-P and 74% of NP knees, *p* = 0.043). Patellar tendon hyperemia was more common in H + P knees (52%, vs. 19% among H-P and 23% among NP, *p* = 0.025). Participants who reported taking scheduled non-steroidal anti-inflammatory drugs (NSAIDs) had an increased risk of synovial effusion (RR = 1.83, 95% CI = 1.07–2.30, *p* = 0.026) and a trend towards increased risk of a higher synovial effusion/hypertrophy quantitative score (RR = 1.77, 95% CI = 0.92–3.38, *p* = 0.086).

**Conclusions:**

While positive MSUS findings were frequent in all participants, patellar tendon hyperemia was more frequent in participants with knee hypermobility and pain symptoms. Additionally, reported use of NSAIDs was associated with an increased risk of synovial effusion and higher synovial effusion/hypertrophy quantitative score. Further study should assess correlation between tendon abnormalities and degree of pain symptoms as well as the effect of NSAIDs on MSUS findings.

## Background

Musculoskeletal pain is a common symptom in children and adolescents. The symptom becomes more prevalent as age increases [1] and is a frequent reason for evaluation in both primary and specialty care [2]. Joint hypermobility is a risk factor for musculoskeletal pain in adolescents, particularly in certain joints such as the knee [3, 4]. The prevalence of joint hypermobility varies in different populations but can affect over 30% of children and adolescents [5]. The pathophysiology of joint hypermobility contributing to joint pain is unclear but may be related to excess movement leading to stress and micro-trauma [2]. Given the chronicity of the pain and the common delay in diagnosis of joint hypermobility [6, 7], children are often referred to pediatric rheumatology and other subspecialists for evaluation of joint pain.

Musculoskeletal ultrasound (MSUS) use is increasing within pediatric medicine, particularly within pediatric rheumatology [8, 9]. MSUS is shown to be more sensitive than physical examination in detecting active synovitis and subclinical arthritis [10, 11]. Additionally, MSUS can reveal findings of tendon abnormalities and enthesitis [12, 13]. To date, the majority of MSUS literature focuses on findings in healthy children and those with inflammatory arthritis, particularly juvenile idiopathic arthritis (JIA). Few studies exist regarding tendon measurements or elastography on ultrasound imaging in populations with joint hypermobility [14, 15]. However, these studies include mostly adult participants and report minimal MSUS views of the joints.

It is important to describe the MSUS findings in joint hypermobility to potentially differentiate from other causes of chronic joint pain such as inflammatory arthritis, apophysitis, and tendinopathies. Specific abnormal findings from joint hypermobility could help with additional diagnostic or therapeutic decisions. Additionally, abnormal findings could provide further insight into the pathophysiology of pain associated with joint hypermobility.

Our aim is to describe the MSUS characteristics of the knee in a population with joint hypermobility and pain in comparison to asymptomatic children with and without joint hypermobility.

## Methods

### Participant recruitment

Participants were recruited and assigned by knee into three groups for a case-control study: case group with knee joint hypermobility and pain symptoms (H + P), control group with knee joint hypermobility without pain symptoms (H-P), and control group with no hypermobility or pain (NP). Participants in the case group were recruited from a pediatric rheumatology clinic with a multidisciplinary clinic dedicated to patients with joint hypermobility. Participants in the control groups were recruited from the same pediatric rheumatology clinic as well as a pediatric ophthalmology clinic and a pediatric dermatology clinic at an academic children’s hospital. Ethics approval was obtained from the Nationwide Children’s Institutional Review Board (IRB18–01176). Participants or their legal guardians if participants were under 18 years of age provided written informed consent.

Inclusion criteria for the case group were participant age ≥ 14 years, > 10 degrees of knee hyperextension on goniometer measurement per Beighton criteria for hypermobility [16], and pain score ≥ 1 (out of 10) on validated visual pain scale [17] reported by the participant in each knee over the past month. Control participants were matched by age and gender. The H-P control group had knee hypermobility without pain symptoms (pain score = 0). The NP control group had no knee hypermobility (≤10 degrees of knee hyperextension) and no pain symptoms. A single physician performed goniometry of Beighton criteria for all participants. Participants in any group were excluded if they had a current diagnosis associated with arthritis, history of previous knee surgery, or history of knee trauma within the month prior to evaluation. Participant body mass index (BMI) and non-steroidal anti-inflammatory drug (NSAID) use were collected from the medical record.

### Ultrasound assessment

Participants had MSUS assessment of one or both knees, depending on their hypermobility measurements, pain symptoms and exclusion criteria. For example, if a participant had previous unilateral knee surgery, this knee was excluded, but they could enroll in the study for the opposite knee. A pediatric rheumatology fellow trained in MSUS obtained B-mode and Doppler images of each knee according to published guidelines, which include multiple views in the suprapatellar, infrapatellar, medial, lateral and posterior aspects of the knee while the knee is flexed slightly at 30 degrees [18]. MSUS were obtained with GE Logiq S8 equipment with a 4–12-MHz linear array transducer. A blinded pediatric rheumatologist trained in MSUS scored the ultrasound images according to a pediatric scoring system with grading of zero- through three-points on semi-quantitative scale for synovial effusion, hypertrophy and hyperemia [19]. On this scale, zero represents no findings while one through three represent grades of positive findings. The highest score seen on any view of the suprapatellar recess (midline anterior or medial/lateral gutters) of either an effusion or synovial hypertrophy was used as the grade for synovial effusion/hypertrophy quantitative score for each knee. Additionally, images were evaluated for presence or absence of tendon abnormalities, tendon thickness, and cartilage thickness based on previously published definitions [20–22].

### Statistical analysis

Based on power analysis, 33 knees were needed per group for a total of 99 knees to achieve an 80% power to detect a 28% difference in joint effusion rate between the groups, assuming a 60% rate of effusion among healthy knees [23, 24]. As these previous studies have reported variability in knee effusion rates from 60 to 80% in healthy knees, an effect size of greater than 20% was necessary to represent a clinically meaningful difference. Descriptive statistics are reported at the participant level for overall demographic variables and NSAID use. Descriptive statistics are reported at the knee level for all MSUS findings, pain scores, and any measurement that can vary between a participant’s knees. Variables are compared by knee type using logistic, Poisson, and linear mixed effects models depending on the variable distribution. Comparisons are not made for variables with an event rate of less than five. Poisson generalized estimating equations with robust standards errors are used to evaluate the association of knee type with synovial effusion and synovial effusion/hypertrophy quantitative score while accounting for NSAID use and BMI. All analyses were conducted using SAS 9.4 (SAS Institute, Cary, NC).

## Results

Fifty participants enrolled in the study, and MSUS was completed on 91 knees. Eight knees are missing from the control groups to achieve the desired study power as recruitment ended earlier than expected due to COVID-19 pandemic and subsequent restrictions placed on research recruitment.

### Participant characteristics

Participant characteristics are summarized in Table 1 by group. Three participants had one knee enrolled in the H + P group and one knee enrolled in the H-P group. Male participants accounted for about one-quarter of the population. BMI was not significantly different between the groups. NSAID use, either scheduled or as needed, was more common in participants of the H + P knees (63% vs. 18% of H-P and 13% of NP participants, *p* < 0.001).

**Table 1:** Participant Characteristics

**Characteristics by participant**	**All (*n*** **= 50)**	**H + P (*n*** **= 20)**	**H-P (*n*** **= 16)**	**NP (*n*** **= 17)**	***p*-value**
Male, n (%)	12 (24)	8 (24)	8 (30)	8 (26)	0.892
Age (years), mean (sd)	16.1 (1.4)	16.2 (1.2)	16.0 (1.4)	16.2 (1.5)	0.764
BMI, mean (sd)	26.1 (6.7)	26.7 (6.9)	26.0 (5.5)	25.5 (7.5)	0.762
Pain Score, mean (sd)	.	4.6 (1.5)	.	.	
Beighton Score, mean (sd)	3.9 (3.1)	5.8 (2.3)	5.1 (2.7)	0.7 (0.9)	**< 0.001**
Knee measurement (degrees), mean (sd)	189.6 (4.9)	192.9 (1.9)	192.7 (1.8)	183.3 (2.3)	**< 0.001**
**Characteristics by knee**	**All (*n*** **= 91)**	**H + P (*n*** **= 33)**	**H-P (*n*** **= 27)**	**NP (*n*** **= 31)**	***p*-value**
NSAID use, n (%)					**< 0.001**
None	61 (67)	12 (36)	22 (81)	27 (87)	
As needed	18 (13)	11 (33)	3 (11)	4 (13)	
Scheduled	12 (13)	10 (30)	2 (7)	0 (0)	

Legend: Significant *p*-values appear in bold font. Three participants had one knee enrolled in the H + P group and one knee enrolled in the H-P group. *H + P* hypermobility with pain group, *H-P* hypermobility without pain group, *NP* no hypermobility or pain group, *BMI* body mass index, *NSAID* non-steroidal anti-inflammatory drug

As expected, based on study design, NP knees had a significantly lower Beighton score compared to H + P or H-P knees, while there was no difference in Beighton score among H + P and H-P groups. Similarly, NP knees had less knee hyperextension by goniometry than H + P or H-P knees, while there was no difference among H + P and H-P groups.

### Ultrasound findings

**Fig. 1:**
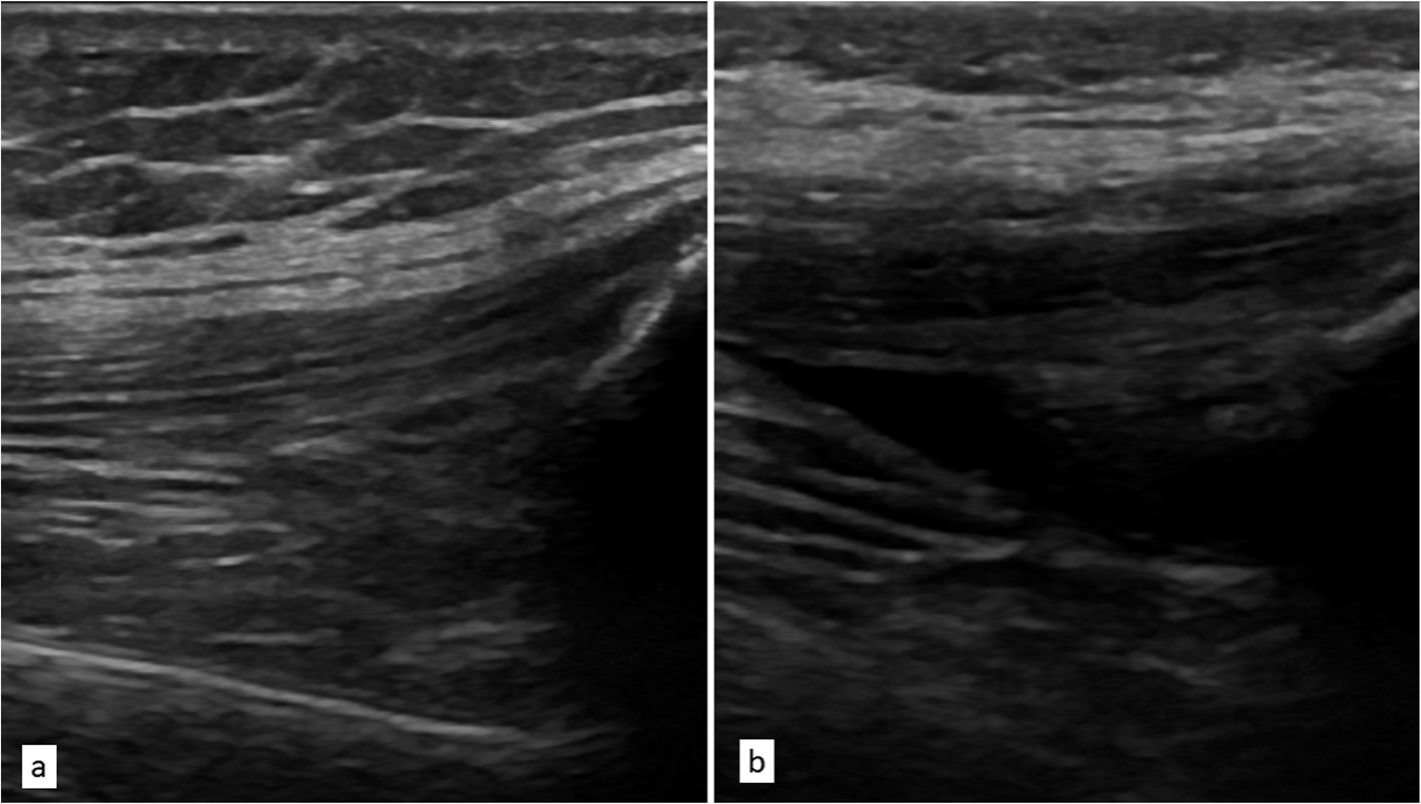
Suprapatellar longitudinal images of participant knees in B-mode. Legend: **a**. Synovial effusion/hypertrophy quantitative score of zero. **b**. Synovial effusion/hypertrophy quantitative score of two

MSUS findings are summarized in Table 2 by group. H + P knees were more likely to have any type of positive finding noted on MSUS compared to control groups (94% vs. 70% of H-P and 74% of NP knees, *p* = 0.043), however, all three groups had a high percentage of positive findings. Over half of the knees in all groups had some degree of synovial effusion. Effusion was most common within the lateral parapatellar synovium in all three groups (42% of H + P, 33% of H-P, and 42% of NP knees) compared to the medial parapatellar (33% of H + P, 25% of H-P, and 32% of NP knees) and suprapatellar synovium (21% of H + P, 19% of H-P, and 19% of NP knees). The synovial effusion/hypertrophy quantitative score did not differ among the groups (1.09 for H + P, 0.78 for H-P, and 0.97 for NP, *p* = 0.488). Figure 1 shows representative examples from study participants of different synovial effusion/hypertrophy quantitative scores. The number of participants with quantitative score of two or greater on the scale of zero to three was similar amongst the groups (24% of H + P, 15% of H-P, and 27% of NP knees). Three knees in the NP group received a score of three for large effusion and/or significant synovial hypertrophy.

**Table 2:** Ultrasound Findings

	All (***n*** = 91)	H + P (***n*** = 33)	H-P (***n*** = 27)	NP (***n*** = 31)	***p***-value
Synovial Effusion, n (%)	52 (57)	21 (64)	14 (52)	17 (55)	0.714
Synovial Hypertrophy, n (%)	18 (20)	7 (21)	4 (15)	7 (23)	0.715
Synovial Effusion/Hypertrophy Quant Score^a^, mean (sd)	1.1 (0.9)	1.1 (0.9)	0.8 (0.7)	1.0 (1.0)	0.488
Synovial Hyperemia, n (%)	2 (2)	1 (3)	1 (4)		
Quadriceps Tendon Edema, n (%)	9 (10)	6 (18)	0 (0)	3 (10)	0.070
Quadriceps Tendon Hyperemia, n (%)	3 (3)	1 (3)	1 (4)	1 (3)	1.000
Quadriceps Tendon Tear, n (%)	1 (1)	1 (3)			
Quadriceps Tendon Enthesitis, n (%)	1 (1)	1 (3)			
Quad Tendon Width, Proximal, mm, mean (sd)	5.1 (1.0)	5.0 (0.9)	5.0 (0.7)	5.5 (1.1)	0.144
Quad Tendon Width, Distal Insertion, mm, mean (sd)	5.6 (0.8)	5.5 (0.8)	5.6 (0.8)	5.7 (0.9)	0.680
Patellar Tendon Edema, n (%)	4 (4)	3 (9)	1 (3)		
Patellar Tendon Hyperemia, n (%)	29 (32)	17 (52)	5 (19)	7 (23)	**0.025**
Patellar Tendon Tear, n (%)	0 (0)				
Patellar Tendon Enthesitis, n (%)	0 (0)				
Patellar Tendon Width, Proximal Origin, mm, mean (sd)	4.1 (0.8)	4.1 (0.7)	4.2 (1.0)	4.0 (0.8)	0.546
Patellar Tendon Width, Distal Insertion, mm, mean (sd)	4.60 (0.7)	4.5 (0.6)	4.6 (0.7)	4.7 (0.7)	0.810
Pat Tendon Width, Smallest Central, mm, mean (sd)	3.0 (0.5)	2.9 (0.4)	3 (0.5)	3.0 (0.5)	0.817
Cartilage Thickness, mm, mean (sd)	2.6 (0.7)	2.9 (0.7)	2.4 (0.5)	2.6 (0.7)	0.414
Any Finding, n (%)	73 (80)	31 (94)	19 (70)	23 (74)	**0.043**

Legend: Significant *p*-values appear in bold font. ^a^Synovial effusion/hypertrophy quantitative score is based on a zero to three-point Likert scale. *H + P* hypermobility with pain group, *H-P* hypermobility without pain group, *NP* no hypermobility or pain group

**Fig. 2:**
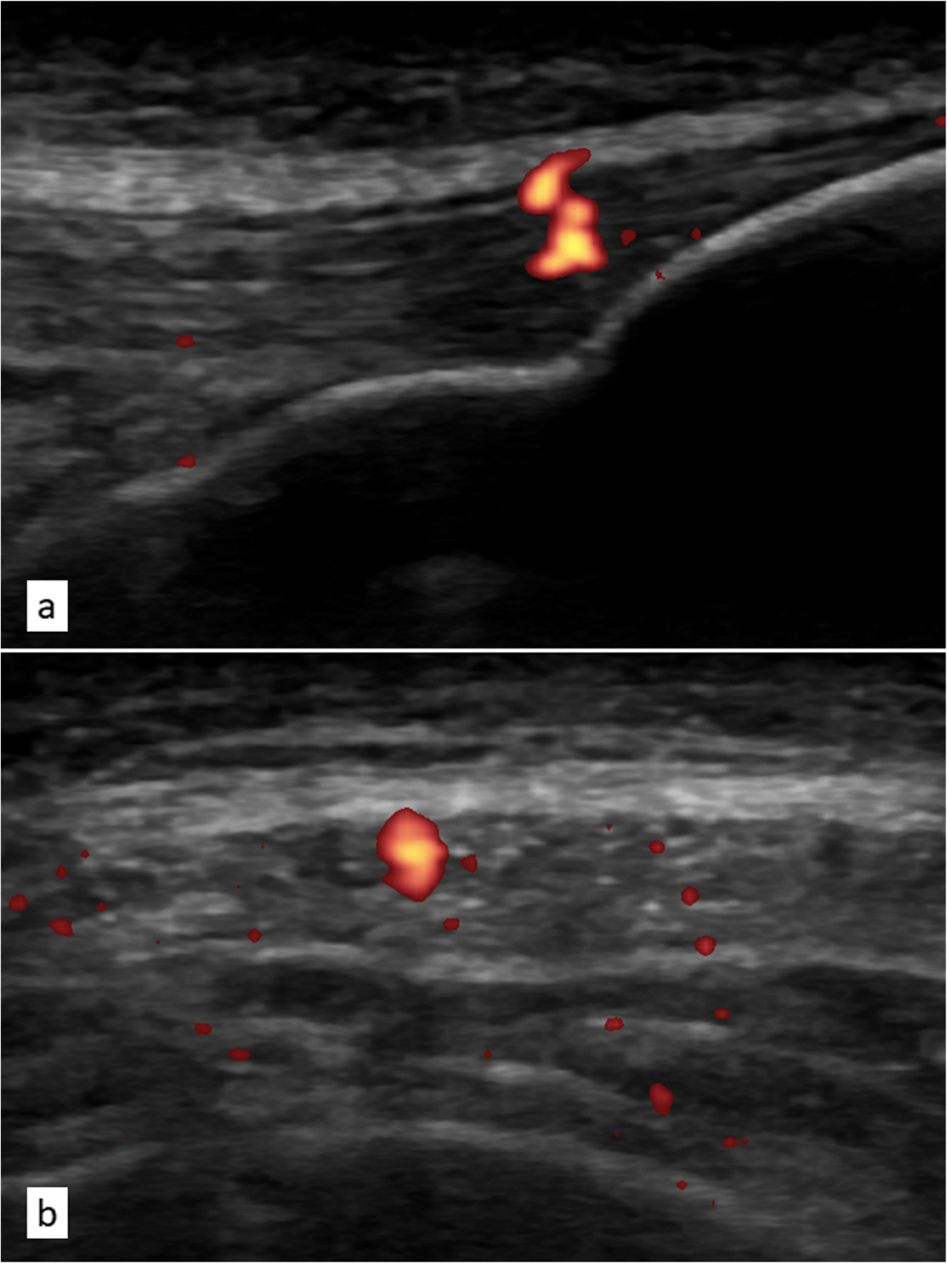
Orthogonal infrapatellar images of the distal patellar tendon with positive Doppler findings. Legend: **a**. Longitudinal image. **b**. Transverse image

Patellar tendon hyperemia was a more common finding in H + P knees (52% vs. 19% of H-P and 23% of NP knees, *p* = 0.025). Figure 2 shows an example of positive findings in the patellar tendon of a study participant. Quadriceps tendon edema was also noted in more H + P knees (18% vs. 0% of H-P and 10% of NP knees, *p* = 0.070). Tendon thickness did not differ between the groups at any location for the quadriceps or patellar tendons. Cartilage thickness of the distal femur did not differ between the groups.

### Relative risk analyses

Hypermobility, BMI, and NSAID use were evaluated as risk factors for any amount of synovial effusion (Table 3) and synovial effusion/hypertrophy quantitative score greater than zero (Table 4) in univariable and multivariable analyses. Hypermobile knees were not associated with an increased risk of effusion or synovial effusion/hypertrophy quantitative score. Participants on scheduled NSAIDs had an 83% greater risk of effusion (adjusted relative risk = 1.83, *p* = 0.026) and 77% greater risk of a higher synovial effusion/hypertrophy quantitative score (adjusted relative risk = 1.77, *p* = 0.086).

**Table 3:** Risk of Any Synovial Effusion

	Univariate			Multivariable		
	RR	95%CI	*p*-value	RR	95%CI	*p*-value
H + P	1.16	(0.73,1.85)	0.534	0.91	(0.52, 1.57)	0.724
H-P	0.95	(0.54, 1.65)	0.844	0.90	(0.52, 1.59)	0.720
NP	reference		reference	reference		reference
BMI	1.00	(0.97,1.03)	0.948	0.99	(0.96, 1.02)	0.431
No NSAIDs	reference		reference	reference		reference
NSAIDs as needed	1.20	(0.73,1.94)	0.470	1.33	(0.76, 2.30)	0.317
Scheduled NSAIDs	1.64	(1.14, 2.36)	**0.008**	1.83	(1.07, 2.30)	**0.026**

Legend: Significant *p*-values appear in bold font. *H + P* hypermobility with pain group, *H-P* hypermobility without pain group, *NP* no hypermobility or pain group, *BMI* body mass index, *NSAID* non-steroidal anti-inflammatory drug

**Table 4:** Risk of Synovial Effusion/Hypertrophy Quantitative Score Greater Than Zero

	Univariate			Multivariable		
	RR	95%CI	*p*-value	RR	95%CI	*p*-value
H + P	1.13	(0.64, 1.99)	0.680	0.89	(0.42, 1.87)	0.753
H-P	0.80	(0.46, 1.40)	0.441	0.76	(0.44, 1.33)	0.337
NP	reference		reference	reference		reference
BMI	1.02	(0.99, 1.05)	0.292	1.01	(0.98, 1.03)	0.490
No NSAIDs	reference		reference	reference		reference
NSAIDs as needed	1.20	(0.64, 2.24)	0.575	1.13	(0.55, 2.34)	0.737
Scheduled NSAIDs	1.79	(1,12, 2.87)	**0.015**	1.77	(0.92, 3.38)	0.086

Legend: Significant *p*-value appears in bold font. *H + P* hypermobility with pain group, *H-P* hypermobility without pain group, *NP* no hypermobility or pain group, *BMI* body mass index, *NSAID* non-steroidal anti-inflammatory drug

## Discussion

To the best of our knowledge, our study represents the first description of a comprehensive MSUS assessment of the knee in participants with joint hypermobility. The study identified several notable findings. Patellar tendon hyperemia was more common in the knees with hypermobility and pain symptoms. MSUS findings were frequent in all participants, however, participants with knee hypermobility and pain had significantly more positive findings. Synovial effusion was the most common finding in all three groups but was found with similar frequency amongst the groups. NSAID use was associated with an increased risk of synovial effusion and increased synovial effusion/hypertrophy quantitative score.

In previous reports of knee tendon features on MSUS in healthy children, no vascularity was detected in the patellar tendons [25, 26]. Patellar tendon hyperemia is a significantly different finding in our case participants compared to control groups, which suggests that the tendon may be associated with pathology in hypermobile joints. Patellar tendon thickness at the proximal patellar ligament is similar in our whole study cohort to reports of tendon thickness in healthy children of similar ages (4.1 mm vs 3.5 mm [25] vs 4.0 mm [27] at the proximal patellar ligament). However, patellar tendon thickness at the distal patellar ligament is larger in our study compared to other reports in healthy children (5.6 mm vs 3.5 mm [26] vs 3.7 mm [27]). Patellar tendon thickness was not significantly different between our case and control groups, so further investigation will be necessary to assess this finding of a difference in tendon thickness. Additionally, larger and longitudinal studies are necessary to further describe how these findings relate to degree of pain symptoms and how the findings change over time with symptoms.

The frequency of knee synovial effusion by MSUS in healthy children has been reported from 60 to 80% depending on age-group [23, 24]. In a German population of healthy children, the frequency of suprapatellar synovial effusions on MSUS in participants between ages 13 and 18 years was between 60 and 70% [23], which is slightly higher than our percentage of control participants with synovial effusion in any space (suprapatellar, medial, lateral). Our percentage of case group participants with synovial effusion (64%) is similar to the frequency of asymptomatic effusions in the German cohort. This similarity highlights that positive findings on MSUS do not necessarily correlate to underlying pathology. However, we investigated the medial and lateral parapatellar spaces in addition to the suprapatellar space which makes our study more sensitive for detecting any effusion. Additionally, the number of knees with higher synovial effusion/hypertrophy quantitative scores did not differ between the groups. It is likely that many of the participants in the H + P case group have physiologic fluid on MSUS rather than abnormal or pathologic fluid that correlates with their pain symptoms. Three knees in the NP group received the highest quantitative score for synovial effusion/hypertrophy so the degree of positive findings was not able to differentiate the knees. The scoring tool [19] which was developed for patients with juvenile idiopathic arthritis may not be applicable to differentiate findings amongst this population.

Interestingly, use of NSAIDs was associated with an increased risk of synovial effusion and higher synovial effusion/hypertrophy quantitative score, even after accounting for participants’ knee grouping. We suggest two potential explanations of this result as we would not expect for NSAIDs to increase synovial effusion or hypertrophy findings. NSAIDs are utilized as a therapy for inflammatory arthritis where there may be findings of synovial effusion, hypertrophy or hyperemia on MSUS [28], and these findings can be improved or masked with NSAID therapy [29]. In our study, NSAID use was significantly higher in participants from the case group. This medication may have been treating some of their MSUS findings, and if they were not taking the medication, more MSUS findings may have been present. Alternatively, scheduled NSAIDs may have partially treated symptoms and MSUS findings of an inflammatory arthritis. While none of the patients had received a diagnosis of inflammatory arthritis at the time of the study, the participants were not followed longitudinally after the study to see if their diagnosis changed over time. As a second potential explanation, the case group participants were recruited from a multi-disciplinary clinic for patients with joint hypermobility in which NSAIDs are a commonly prescribed medication for pain (per verbal discussion). If participants were recruited from primary or other specialty care clinics without this prescribing practice, the association of NSAID use and increased risk of synovial effusion and synovial effusion/hypertrophy quantitative score may not be significant. Lastly, the NSAID use variable was collected from review of the medical record. Participants may not have been taking NSAID as prescribed or may not have reported any over the counter NSAID use for documentation in the medical record. Future study should evaluate MSUS findings in comparison to actual NSAID use as well as potential subsequent diagnoses of inflammatory arthritis.

This study has several limitations. The number of participants is relatively low, and recruitment did not achieve the desired enrollment based on power calculations due to early recruitment conclusion for COVID-19 pandemic. A single pediatric rheumatology fellow obtained the MSUS images, and a single pediatric rheumatologist scored the images so these results may not be reproducible in an evaluation by multiple providers. However, the pediatric rheumatologist was blinded to the participant group and clinical information, and having a single reviewer eliminates potential for inter-rater variability. Additionally, all imaging parameters were scored with the knee flexed to 30 degrees, and while this is typical for assessment of joint space pathology there may have been some hyperemia of the tendons overlooked as this is best evaluated with the tendons maximally relaxed. Lastly, case participants were recruited from a single sub-specialty clinic so their findings may not be reflective of symptomatic knee hypermobility in the general population.

## Conclusions

Our results suggest that the tendons may be an area of increased pathology in a population with knee hypermobility and pain symptoms. This population had frequent findings on MSUS, but all findings may not correlate with abnormal pathology as they also occur in participants without pain symptoms and without hypermobility findings. Positive findings occurred in many of the MSUS views which highlights the importance of a comprehensive MSUS assessment. Further study is needed to evaluate for MSUS findings and causation of pain symptoms.

## Data Availability

The datasets used and/or analyzed during the current study are available from the corresponding author [LRB] on reasonable request. The data are not publicly available as this information could compromise the privacy of research participants.

## Abbreviations

BMI: Body mass index
H + P: Hypermobility and pain symptoms
H-P: Hypermobility without pain symptoms
JIA: Juvenile idiopathic arthritis
MSUS: Musculoskeletal ultrasound
NP: No hypermobility or pain symptoms
NSAIDs: Non-steroidal anti-inflammatory drugs
